# ^99m^Tc-NTP 15-5 assessment of the early therapeutic response of chondrosarcoma to zoledronic acid in the Swarm rat orthotopic model

**DOI:** 10.1186/2191-219X-3-40

**Published:** 2013-05-20

**Authors:** Elisabeth Miot-Noirault, Emmanuelle David, Aurélien Vidal, Caroline Peyrode, Sophie Besse, Marie-Mélanie Dauplat, Marie-Françoise Heymann, François Gouin, Jean-Michel Chezal, Dominique Heymann, Françoise Rédini

**Affiliations:** 1 INSERM UMR 990, Université d’Auvergne, BP 184, Rue Montalembert, Clermont-Ferrand, Cédex, 63005, France; 2 INSERM UMR 957, Nantes, 44000, France; 3 Laboratoire de Physiopathologie de la Résorption Osseuse et Thérapie des Tumeurs Osseuses Primitives, Faculté de Médecine, Nantes Atlantique Universités, Université de Nantes, Nantes, 44000, France; 4 Département d’anatomo-pathologie, Centre Jean Perrin, Clermont-Ferrand, 63001, France; 5 Département d’anatomo-pathologie, Hôtel Dieu, CHU de Nantes, Nantes, 44000, France; 6 Service d’orthopédie, Hôtel Dieu, CHU de Nantes, Nantes, 44000, France

**Keywords:** Chondrosarcoma, Zoledronic acid, ^99m^Tc-NTP 15-5 radiotracer, Proteoglycans

## Abstract

**Background:**

Since proteoglycans (PGs) appear as key partners in chondrosarcoma biology, PG-targeted imaging using the radiotracer ^99m^Tc-*N*-(triethylammonium)-3-propyl-[15]ane-N5 (^99m^Tc-NTP 15-5) developed by our group was previously demonstrated to be a good single-photon emission computed tomography tracer for cartilage neoplasms. We therefore initiated this new preclinical study to evaluate the relevance of ^99m^Tc-NTP 15-5 imaging for the *in vivo* monitoring and quantitative assessment of chondrosarcoma response to zoledronic acid (ZOL) in the Swarm rat orthotopic model.

**Findings:**

Rats bearing chondrosarcoma in the orthotopic paratibial location were treated by ZOL (100 μg/kg, subcutaneously) or phosphate-buffered saline, twice a week, from day 4 to day 48 post-tumor implantation. ^99m^Tc-NTP 15-5 imaging was performed at regular intervals with the target-to-background ratio (TBR) determined. Tumor volume was monitored using a calliper, and histology was performed at the end of the study. From day 11 to day 48, mean TBR values ranged from 1.7 ± 0.6 to 2.3 ± 0.6 in ZOL-treated rats and from 2.1 ± 1.0 to 4.9 ± 0.9 in controls. Tumor growth inhibition was evidenced using a calliper from day 24 and associated to a decrease in PG content in treated tumor tissues (confirmed by histology).

**Conclusions:**

This work demonstrated two proofs of concept: (1) biphosphonate therapy could be a promising therapeutic approach for chondrosarcoma; (2) ^99m^Tc-NTP 15-5 is expected to offer a novel imaging modality for the *in vivo* evaluation of the extracellular matrix features of chondrosarcoma, which could be useful for the follow-up and quantitative assessment of proteoglycan ‘downregulation’ associated to the response to therapeutic attempts.

## Findings

### Introduction

Considering the 10-year survival rate of chondrosarcoma (from 29% to 83% depending on the subtype and grading), improving its clinical management is a challenge and novel therapeutic approaches are needed [1]. This implies that *in vivo* markers of the biologic phenotypic features of chondrosarcoma behavior in the bone environment could be used as quantitative criteria of grading, follow-up, and early evaluation of response to therapy.

Since proteoglycans (PGs) appear as key partners in chondrosarcoma biology, PG-targeted imaging using the radiotracer ^99m^Tc-*N*-(triethylammonium)-3-propyl-[15]ane-N5 (^99m^Tc-NTP 15-5) developed by our group was expected to be a good single-photon emission computed tomography (SPECT) tracer for the molecular imaging of cartilage neoplasms in nuclear medicine [2,3].

Resistance to chemotherapy is well known to make chondrosarcoma therapeutic management difficult [4]. The bisphosphonate zoledronic acid (ZOL) has demonstrated its therapeutic interest in a variety of tumors affecting the bones, such as osteosarcoma, Ewing's sarcoma, and chondrosarcoma [5-7].

This study was therefore initiated in the well-characterized Swarm rat chondrosarcoma (SRC) orthotopic model to determine the relevance of ^99m^Tc-NTP 15-5 imaging for an early quantitative characterization of chondrosarcoma response to ZOL.

## Methods

Protocols were performed under the authorization of the French Directorate of Veterinary Services (Authorization No. C63-113-10) and were conducted under the supervision of authorized investigators in accordance with the institution’s recommendations for the use of laboratory animals.

### Model

Experiments were conducted on 24 male Sprague Dawley rats (Charles River, L'Arbresle, France). The SRC model was a generous gift from Dr. P.A. Guerne (Switzerland) as tissue fragments, which were frozen until use. Allograft transplantation of a 10-mm^3^ SRC fragment was performed on the right hindlimb in the paratibial location of the anesthetized animals (isoflurane (Abbott, Rungis, France) in air (1.5%, 1 L/min)) associated with an intramuscular injection of 100 mg/kg of ketamine (Imalgene®, Rhone Merieux, Lyon, France), as published [3,6,8].

### ZOL treatment

ZOL was kindly provided as a research-grade disodium salt by Novartis Pharma AG (Basel, Switzerland). Rats were randomly divided into two groups:

1. ZOL-treated: subcutaneous administration of 100 μg/kg twice a week from day 4 to day 48 after implantation (*n* = 12)

2. Control: PBS in the same conditions (*n* = 12)

### Tumor growth assessment

Tumor volume was calculated from calliper measurement of the largest (*L*) and smallest (*S*) perpendicular tumor diameters, using the formula: *V*_(mm3)_ = 0.5 × *L* × *S*^2^[3]. Data were expressed as mean ± standard deviation. At each time point, the tumor volumes of ZOL and controls were compared by analysis of variance (ANOVA) with a level of significance set at *p* < 0.05.

### ^99m^Tc-NTP 15-5 imaging

^99m^Tc-NTP 15-5 imaging was performed at days 4 (before treatment), 11, 16, 24, 36, and 48 after implantation. NTP 15-5 was radiolabeled with ^99m^Tc as described [3].

Acquisitions (10-min duration, 15% window at 140 keV) of each hindlimb of anesthetized animals positioned over a 10-cm collimator of a small-animal gamma camera (γImager, Biospace, Paris, France) were performed, 30 min after i.v. administration of 30 MBq of radiotracer. Fixed-size regions of interest (ROIs) were delineated over the tumor, adjacent muscles, and contralateral muscles, and average counts (cpm/mm^2^) were obtained. The use of activity profile for ROI placement ensured easy, reproducible positioning of the ROI for serial images.

At each time point and for each animal, the target-to-background ratio (TBR) was calculated as follows: TBR = Average count in the tumor (cpm/mm^2^) / Average count in the contralateral muscle (cpm/mm^2^). Data were expressed as mean ± standard deviation.

Analysis of data with multiple comparisons was performed by repeated-measures ANOVA followed by Tukey’s post-test, with a level of significance set at *p* < 0.05.

### Histology

At the end of the study, tumors fixed in 4% formalin were embedded in paraffin, sectioned (5 μm), and stained with hematoxylin-eosin and alcian blue (AB).

## Results

### ZOL inhibited tumor growth

**Figure 1:**
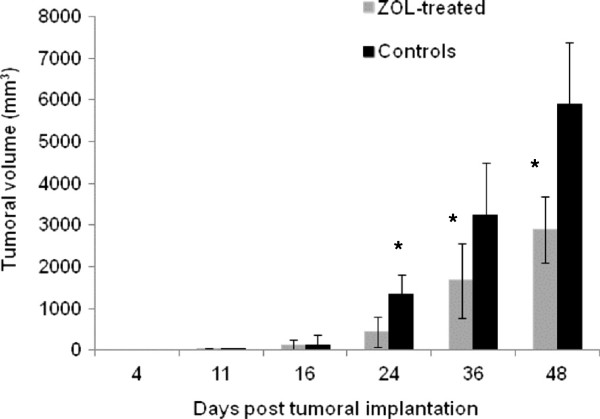
**Representative time course of the mean tumor volume ± standard deviation of ZOL-treated and control groups.** Both groups have *n* = 12 animals. The asterisk indicates *p* < 0.05.

As shown in Figure 1, ZOL inhibited tumor growth as compared to controls: From day 24 to day 48, a significant (*p* < 0.05) difference in tumor volume was observed between control and treated groups.

**Figure 2:**
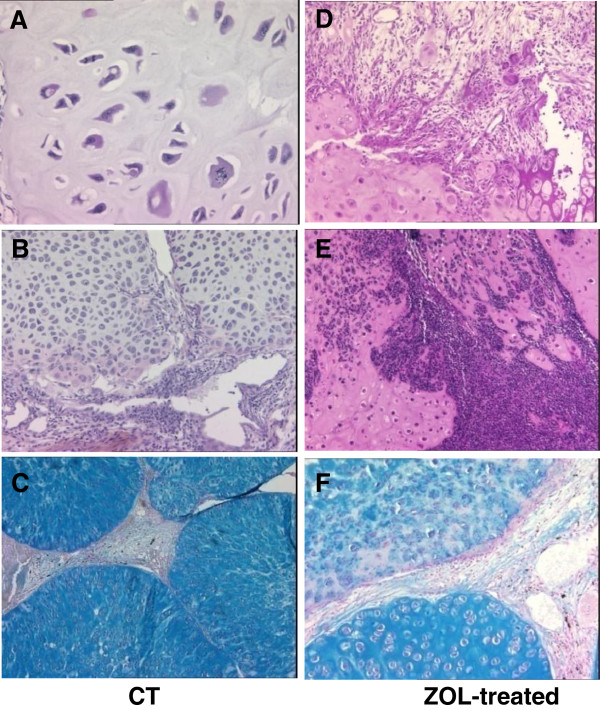
**Histological analysis of tumor tissue at the end of the study: control (A to C) and ZOL-treated (D to F) groups.** The tumor slices were stained with hematoxylin-eosin (**A**, **B**, **D**, **E**) or with alcian blue (**C**, **F**). Magnification: ×50 (**C**), ×100 (**B**, **D**, **E**, **F**), and ×200 (**A**). CT, controls; ZOL, zoledronic acid.

Tumor regression patterns were clearly evidenced by histology (Figure 2): whereas controls showed nuclear atypia, anisokaryosis, mitosis, lobular arrangement of the tumor, and metachromasia (Figure 2A,B,C), ZOL-treated animals demonstrated fibrous and inflammatory remodeling patterns of tumor regression (Figure 2D,E). Metachromasia, reflecting PG content, was also decreased in ZOL-treated animals as compared to controls (Figure 2F versus Figure 2C).

### ^99m^Tc-NTP 15-5 monitoring of in vivo chondrosarcoma response to ZOL

**Figure 3:**
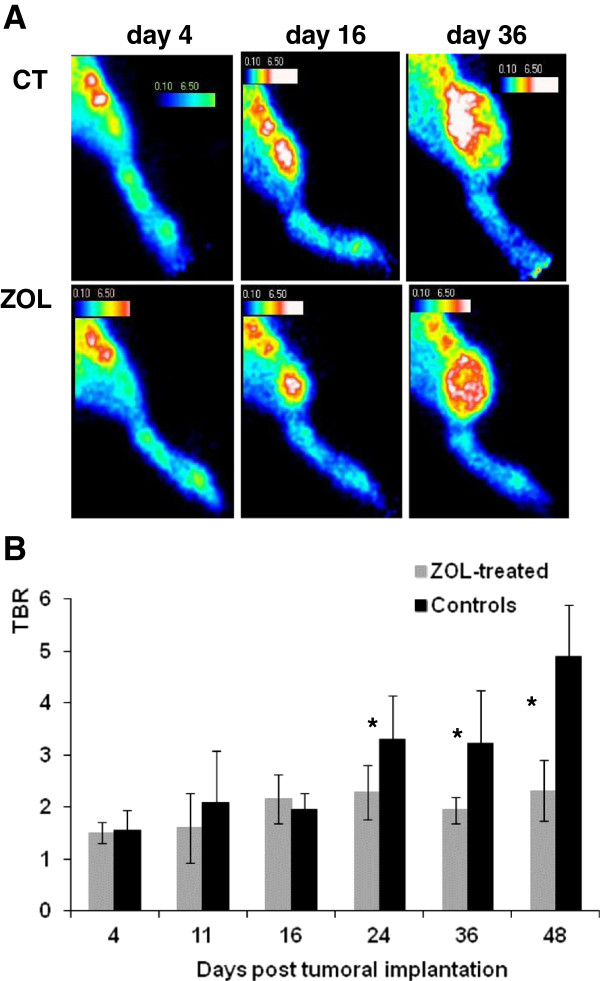
^**99m**^**Tc-NTP 15-5 imaging of control and ZOL-treated groups.** (**A**) Representative *in vivo* scintigraphic images of the tumor-bearing hindlimb obtained for controls and ZOL-treated animals at various stages of the study. (**B**) Semi-quantitative analysis of ^99m^Tc-NTP 15-5 imaging: results are expressed as mean TBR values ± standard deviation. CT, controls; ZOL, zoledronic acid-treated; TBR, target-to-background ratio. The asterisk indicates *p* < 0.05.

As illustrated in Figure 3A, for representative animals, ^99m^Tc-NTP 15-5 accumulation was observed in the joints and at the sites of tumor implantation as early as day 4. As pathology evolved, a weaker radiotracer accumulation was observed in ZOL-treated tumors as compared to controls.

From day 11 to day 48, mean TBR values ranged from 1.7 ± 0.6 to 2.3 ± 0.6 in ZOL-treated rats and from 2.1 ± 1.0 to 4.9 ± 0.9 in controls (Figure 3B). Differences in TBR between treated rats and controls were statistically significant (*p* < 0.05) from day 24 to day 48. Repeated-measures ANOVA revealed both a significant ‘between-group effect’ as well as a within-subject effect (group * time).

## Discussion

A few years ago, Heymann's group published the therapeutic relevance of using ZOL in the SRC model [6]. Since then, others have reported the promising effects of bisphosphonates not only on chondrosarcoma proliferation and invasion *in vitro*, but also in clinical practice [7,9,10]. In clinics, chondrosarcoma therapeutic response is still evaluated by conventional imaging by measuring anatomical tumor size reduction [11]. Unlike the late effects of anticancer therapy on tumor size, molecular and metabolic changes are well recognized to be induced much earlier, before morphological changes occur [12].

Our previous study demonstrated ^99m^Tc-NTP 15-5 to be a promising SPECT tracer for the molecular imaging of cartilage neoplasms in nuclear medicine [3]. ^99m^Tc-NTP 15-5 appeared of particular interest since radiotracers currently available in clinics such as ^201^Tl, ^99m^Tc-MIBI, ^99m^Tc-tetrofosmin, ^99m^Tc-DMSA(V), and ^18^F-FDG have evidenced limitations for imaging chondrosarcoma with low cellularity and high chondrogenic matrix [13].

The results reported in this new study bring additional data in favor of ^99m^Tc-NTP 15-5 imaging for the *in vivo* follow-up of chondrosarcoma and more especially the semi-quantitative assessment of therapeutic response *in vivo*, with TBR values being significantly decreased in the treated group with respect to controls from day 24. Chondrosarcoma growth inhibition was confirmed by calliper measurement from day 24 and characterized at the tissue level by histology: interestingly, AB staining evidenced a huge decrease in glycosaminoglycan content in ZOL as compared to controls. Such PG ‘downregulation’ reflects the tumor proliferation inhibition at the extracellular matrix level and is expected to be at the origin of the decreased accumulation of the ^99m^Tc-NTP 15-5 radiotracer in tumors responding to ZOL.

## Conclusion

^99m^Tc-NTP 15-5 is expected to offer a novel imaging modality for the semi-quantitative evaluation of PG *in vivo* as markers of the extracellular matrix features of chondrosarcoma, which could be useful for the follow-up and evaluation of the response to therapeutic attempts. Combining chondrosarcoma functional imaging at the PG level with relevant targeted therapies may represent the opportunity to bridge the gap between preclinical and clinical testing, which is of real need for improving the management of this pathology.

## Competing interests

The authors declare that they have no competing interests.

## Authors’ contributions

EMN, CP, and SB carried out the experimental treatments and imaging studies. ED and FG carried out the development of the SRC orthotopic model. AV and JMC carried out the synthesis and radiolabeling of NTP 15-5. MMD carried out the histology. MFH participated in the histology analysis. DH and FR conceived of the study and participated in its design and in the manuscript. All authors read and approved the final manuscript.
